# Molecular discrimination of tall fescue morphotypes in association with *Festuca* relatives

**DOI:** 10.1371/journal.pone.0191343

**Published:** 2018-01-17

**Authors:** Shyamal K. Talukder, Perumal Azhaguvel, Konstantin Chekhovskiy, Malay C. Saha

**Affiliations:** Noble Research Institute, LLC, Ardmore, OK, United States of America; Saint Mary's University, CANADA

## Abstract

Tall fescue (*Festuca arundinacea* Schreb.) is an important cool-season perennial grass species used as forage and turf, and in conservation plantings. There are three morphotypes in hexaploid tall fescue: Continental, Mediterranean and Rhizomatous. This study was conducted to develop morphotype-specific molecular markers to distinguish Continental and Mediterranean tall fescues, and establish their relationships with other species of the *Festuca* genus for genomic inference. Chloroplast sequence variation and simple sequence repeat (SSR) polymorphism were explored in 12 genotypes of three tall fescue morphotypes and four *Festuca* species. Hypervariable chloroplast regions were retrieved by using 33 specifically designed primers followed by sequencing the PCR products. SSR polymorphism was studied using 144 tall fescue SSR primers. Four chloroplast (NFTCHL17, NFTCHL43, NFTCHL45 and NFTCHL48) and three SSR (nffa090, nffa204 and nffa338) markers were identified which can distinctly differentiate Continental and Mediterranean morphotypes. A primer pair, NFTCHL45, amplified a 47 bp deletion between the two morphotypes is being routinely used in the Noble Research Institute’s core facility for morphotype discrimination. Both chloroplast sequence variation and SSR diversity showed a close association between Rhizomatous and Continental morphotypes, while the Mediterranean morphotype was in a distant clade. *F*. *pratensis* and *F*. *arundinacea* var. *glaucescens*, the P and G1G2 genome donors, respectively, were grouped with the Continental clade, and *F*. *mairei* (M1M2 genome) grouped with the Mediterranean clade in chloroplast sequence variation, while both *F*. *pratensis* and *F*. *mairei* formed independent clade in SSR analysis. Age estimation based on chloroplast sequence variation indicated that the Continental and Mediterranean clades might have been colonized independently during 0.65 ± 0.06 and 0.96 ± 0.1 million years ago (Mya) respectively. The findings of the study will enhance tall fescue breeding for persistence and productivity.

## Introduction

Tall fescue (*Festuca arundinacea* Schreb.) is a perennial, cool-season bunchgrass grown for pasture, hay, silage and turf [1]. It belongs to the genus ‘*Festuca*,’ which is one of the largest genera under the Poaceae family, containing over 500 grass species [2–4]. It is a cross-pollinated allohexaploid and also a member of a polyploid series consisting of a diploid (*F*. *pratensis* Huds.), tetraploid (*F*. *arundinacea var*. *glaucescens Boiss*. hereafter termed *F*. *glaucescens*), octoploid (*F*. *arundinacea subsp*. *atlantigena* [St. Yves] Auquier) and decaploid (*F*. *arundinacea subsp*. *letourneuxiana* St. Yves. [4–6]. Based on morphological and physiological features, three morphotypes (i.e., Continental, Mediterranean and Rhizomatous) have been recognized in hexaploid tall fescue [5]. Most traditional cultivars belong to the Continental type, which is summer active. The Mediterranean type exhibits incomplete summer dormancy with greater growth during fall, but lacks winter hardiness [7, 8]. Rhizomatous morphotypes have mostly been used as turf [9–11]. This group has a longer and more conspicuous rhizome with superior spreading ability [12].

Hand, Cogan [5] suggested that both Continental and Rhizomatous morphotypes originated from the same progenitor species, but through divergent evolution. Various phylogenetic studies, chromosome structures, pairing habits and genomic *in situ* hybridizations inferred diploid meadow fescue (*F*. *pratensis* Huds.) [13, 14] and tetraploid *F*. *glaucescens* [15, 16] as the P and G1G2 genome donors, respectively, for Continental and Rhizomatous tall fescue. Therefore, the genomic constitution of Continental and Rhizomatous tall fescue is denoted PPG1G1G2G2. To the contrary, Mediterranean morphotypes were proposed to have originated from different progenitors [5]. The projection has been substantiated by the development of infertile F_1_’s from Mediterranean X Continental [17–20] and Mediterranean X Rhizomatous crosses [11, 21]. Phylogenetic study [5] and different endophytic fungal associations [22–24] also reinforced the inference of the independent evolution of Mediterranean morphotypes. *F*. *mairei* is a tetraploid fescue with a genome constitution of M1M1M2M2 [25]. It is native to the Mediterranean region, especially the Atlas Mountains of Morocco and into Algeria (http://www.smgrowers.com/products/plants/- plantdisplay.asp?plant_id=3555). Its relationship with other fescue grasses is not well established. However, Borrill [26] proposed the species to be a progenitor of a polyploid *Festuca* predominant in Morocco. Hand, Cogan [5] studied the relationship among three tall fescue morphotypes using rDNA ITS, and nuclear and chloroplast gene sequences. They found a close association between Continental and Rhizomatous morphotypes along with their progenitors, while the Mediterranean morphotype remained isolated and unresolved.

The chloroplast genome has been widely used to describe genetic diversity in plant species. Maternal inheritance and low mutation rate of the chloroplast genome have been considered highly suitable for studying the relationships of various plant species. Duchene and Bromham [27] reported that the substitution rate of the chloroplast genome was correlated with the net diversification rate of the Proteaceae plant family. Along with that, changes in the chloroplast genome were found to be associated with plant speciation [28]. Using chloroplast genome sequences, attempts were made to find universal primers across species for phylogenetic studies [29, 30]. Similarly, SSRs have been frequently used to capture polymorphism and phylogenetic study in many crop species [31].

Persistence and productivity of Continental tall fescue is significantly affected by hot and dry summers in the Southern Great Plains due to its continuous growth habit. Summer dormancy is an important drought avoidance mechanism of Mediterranean morphotypes, which increases the persistence of tall fescue under harsh summer conditions [32]. Understanding and utilization of summer dormancy mechanisms in tall fescue are priority research areas, especially in the context of climate change. Introduction of summer dormancy can ensure sustainable tall fescue production in hot and dry areas. Phenotypic differentiation between Continental and Mediterranean morphotypes is difficult, irrespective of growth chamber, greenhouse or field conditions. Visually, the two morphotypes are not distinguishable and plants need to be exposed under multi-stress condition (cold followed by long day, high temperature and drought) for expression of dormancy. Confounded with drought and high temperature stress, it become very hard for the plants to demonstrate unequivocal occurrence of summer dormancy. Continental fescue also manifests gradual decrease in leaf elongation and ultimately progressive senescence of all mature leaves and eventually dies under severe drought and high temperature stress during summer. It takes long time to measure regrowth which is highly environment dependent and confounded, because without a harsh summer Continentals may survive and show regrowth [33].Thus, identification of molecular markers and/or genomic regions to differentiate the morphotypes would be very helpful to enhance the tall fescue breeding program. Considering all these concerns, this study was undertaken aiming to:

- develop/identify molecular markers to differentiate tall fescue morphotypes, especially Continental and Mediterranean
- evaluate the identified markers for practical application in the breeding program
- study relationship among tall fescue morphotypes along with other *Festuca* species.

## Materials and methods

### Plant materials and DNA extraction

In this study, marker development was undertaken using chloroplast sequence variation and SSR polymorphism. A total of 12 varieties/accessions were used (Table 1). Among those, six were hexaploid tall fescue (3-Mediterraneans, 2-Continentals and 1-Rhizomatous), two diploid meadow fescue (*F*. *pratensis*) and four tetraploid fescue (two *F*. *mairei* and two *F*. *glaucescens*). The plant materials were maintained in Noble Research Institute (NRI) greenhouse using clonal ramets. Leaf tissues were collected and bulked together from several healthy plants of each variety/accession. Genomic DNA was extracted from the freeze-dried samples using Qiagen DNeasy Plant Mini Kit (QIAGEN, Valencia, California), following the manufacturer’s protocols.

**Table 1:** Name, type and genomic information of 12 *Festuca* varieties/accessions used in this study.

SL NO.	Accession/ varieties	Common Name	Species	Type	Ploidy	Genome
1	Ensign	Meadow fescue	*Festuca pratensis* Huds.	Bunch type	2n = 2× = 14	PP
2	Rita	Meadow fescue	*Festuca pratensis* Huds.	Bunch type	2n = 2× = 14	PP
3	PI 283312	Atlas fescue	*Festuca mairei* St. Yves.	Bunch type	2n = 4× = 28	M1M1M2M2
4	PI 283313	Atlas fescue	*Festuca mairei* St. Yves.	Bunch type	2n = 4× = 28	M1M1M2M2
5	PI 289651	Tetraploid fescue	*Festuca arundinacea spp Glaucescens*	Bunch type	2n = 4× = 28	G1G1G2G2
6	PI 289654	Tetraploid fescue	*Festuca arundinacea spp Glaucescens*	Bunch type	2n = 4× = 28	G1G1G2G2
7	Torpedo	Tall fescue	*Festuca arundinacea* Schreb.	Rhizomatous	2n = 6× = 42	PPG1G1G2G2
8	Prosper	Tall fescue	*Festuca arundinacea* Schreb.	Mediterranean	2n = 6× = 42	?
9	Resolute	Tall fescue	*Festuca arundinacea* Schreb.	Mediterranean	2n = 6× = 42	?
10	Flecha Max Q	Tall fescue	*Festuca arundinacea* Schreb.	Mediterranean	2n = 6× = 42	?
11	Texoma MaxQII	Tall fescue	*Festuca arundinacea* Schreb.	Continental	2n = 6× = 42	PPG1G1G2G2
12	KY-31	Tall fescue	*Festuca arundinacea* Schreb.	Continental	2n = 6× = 42	PPG1G1G2G2

### Designing chloroplast-specific primers, PCR amplifications and sequencing

The full-length tall fescue chloroplast genome of 136.048 kb (FJ466687) was downloaded from GenBank [34]. The repetitive region of the genome sequence was identified using BLASTn against all known repeats in the wheat genome database (http://blast.jcvi.org/euk-blast/index.cgi?project=tae1) so that the known repetitive regions could be avoided during primer design. Primers were designed to capture the hypervariable regions in the chloroplast genome by giving a special preference to include intron-exon boundaries within the amplification. Primers were designed using Primer3 software (http://frodo.wi.mit.edu/-primer3/) and synthesized commercially from ThermoFisher Scientific, Waltham, Massachusetts. A total of 48 primer pairs were designed using the tall fescue chloroplast reference genome sequence. The expected size of the PCR products ranged from 453 to 939 bp. The primer list and product size, along with their sequence information is presented in Table A in S1 File.

A touchdown PCR program was designed for all primer sets following the modified protocol of Hand, Cogan [5]. The PCR protocol includes 95°C for 5 min, six cycles of 94°C for 45 sec, 68°C for 5 min decreasing 2°C per cycle, 72°C for 1 min, followed by five cycles of 94°C for 45 sec, 58°C for 2 min decreasing 2°C per cycle, 72°C for 1 min followed by the final 25 cycles of 94°C for 45 sec, 50°C for 1 min, 72°C for 2 min and a final extension of 72°C for 7 min.

From 30 μL PCR products, samples of 5 μL were electrophoresed in 2% agarose gels to confirm the expected bands. After confirmation, the remaining 25 μL PCR products were vacuum purified using the Qiaquick 96 PCR Purification Kit of Qiagen and sequenced using the BigDye terminator in ABI 3730 from Applied Biosystems, Foster City, California. Discrete single band PCR products of the primers in all 12 accessions were separately sequenced using only the reverse primer. After checking the quality of the reads, the poor quality reads were selected and resequenced using the forward primers to obtain good quality reads for further analysis.

### Sequence alignment and calculation of similarity index

Chloroplast sequences obtained from 12 varieties/accessions were assembled and analyzed. Individual sequence reads from each PCR product across all 12 samples were aligned and trimmed manually at both ends to ensure identical and equal sequence read length using SegMan proV8.1 of the Lasergene package (DNASTAR, Inc., Madison, Wisconsin). The individual sequences were then exported as text files. Finally, 33 PCR product sequences of individual samples were combined and formed into a single FASTA file. After trimming and removing the poor quality reads, a final total of 20,056 bp sequence reads from each of the 12 varieties/accessions were aligned by Geneious Pro 5.1.7 using MAFFT v6.814b [35] (Table B in S1 File).

The number of polymorphic loci was counted among the morphotypic groups/species, and a similarity index was calculated using the following formula: Similarity index between two groups = (1- number of polymorphic loci between two groups / total number of polymorphic loci among all the groups).

### SSR genotyping

Tall fescue and meadow fescue ESTs and genomic sequences were used to develop 2,000 SSRs at the NRI. The polymorphism generated using the SSR markers were examined using multiple clonal ramets of Continental (R43-64) and Mediterranean (AGRFA 103–2) tall fescue genotypes. Based on the polymorphic nature, 144 SSRs primers showing clear bands with higher allelic frequencies were selected.

The PCR amplification was performed using GoTaq DNA Polymerase and 5X Colorless GoTaq Reaction Buffer (Promega Corporation, Madison, Wisconsin). The 10 μL PCR mix consisted of 50 mM MgCl_2_, 1.5 mM dNTPs, 0.25 μM forward primer, 1 μM reverse primer, 1 μM fluorescent dye with M13 tail, 0.45 U GoTaq and 10 ng genomic DNA. A single touchdown PCR procedure described earlier was set up for all primers amplification. The PCR reactions were performed in the Gene Amp PCR System 9700 (Applied Biosystems, Foster City, California). The PCR products were analyzed with ABI 3730 DNA Analyzer following the protocol of Mian, Saha [1]. ABI output was converted to a data format by GeneMapper Software v 3.7.

### Morphotype-specific marker identification

The aligned chloroplast sequences were carefully checked and searched for SNPs and indels (insertion and/or deletions). The primers corresponding to the SNPs/indels that discriminated between Mediterranean and Continental morphotypes were selected for further evaluation. All 144 SSR primer amplifications in 12 varieties/accessions were carefully checked by aligning and color coding the amplification results. Primers with consistent polymorphism between Continental and Mediterranean morphotypes were selected for further evaluation.

### Phylogenetic analysis and clade age estimation

Both chloroplast sequence and SSR marker data were independently analyzed to produce phylogenetic trees. Phylogenetic trees were constructed with retrieved chloroplast sequences by Geneious Pro 5.1 following the Tamura-Nei genetic distance model using UPGMA with 1,000 replications. Similarly, FreeTree followed by TreeView were used to ascertain the relationship among the morphotypes and their relatedness with other *Festuca spp*. by using the polymorphism information. For chloroplast sequences, the 167 polymorphic SNPs/indels were converted into a binary matrix. Based on the binary matrix, a genetic similarity matrix was calculated by FreeTree, using UPGMA and Nei and Li/dice coefficients. After calculating the similarity matrix, the phylogenetic relations were displayed schematically as a reference tree in bracketed text form, which was then used in TreeView to display the phylogenetic tree.

The SSR markers were scored and a binary matrix was constructed and transformed to a genetic similarity matrix. The cluster analyses were performed using NTSYSpc version 2.1 (NTSYS-PC 2.10, Applied Biostatistics, Setauket, New York), and the tree was constructed by UPGMA (unweighted pair group method with arithmetic means) with 1,000 replications. Similarly, to confirm the robustness of the phylogenetic result, FreeTree and TreeView softwares were used following the same procedure used for the binary matrix of the chloroplast SNPs/indels.

Polymorphic SNP data generated from the chloroplast sequence were used in Network 4.6.1.3 (http://www.fluxusengineering.com/-sharenet.htm). For age estimation of the morphotypic clades a network was calculated using the median joining method and a default weight of 10. The output file of the network calculation was used to draw the network. The saved network file was then used to calculate age by selecting the complete network parameter with the default mutation rate of 20,180 [36]. The age of the morphotypic clades was estimated by specifying the ancestral and descendant nodes of the network.

### Evaluation and application of morphotype-specific markers

From morphotype-specific markers, four chloroplast and three SSR primers were used to screen 85 tall fescue accessions [24] collected from 15 locations in Greece (Greek collection) for morphotypic differentiation. Each of the 85 accessions were represented by 3–7 genotypes, thus a total of 383 genotypes were scored independently. Relationship analysis was performed independently using chloroplast and SSR primers with the help of FreeTree and TreeView software.

Ten tall fescue plants, representative of an interesting tall fescue population of unknown origin, which has persisted in an Oklahoma field for a long time were collected and screened using the NFTCHL45 primer for morphotype differentiation. DNA was extracted and PCR was performed following the same procedure described earlier, followed by running the PCR product on 1.5% agarose gels. One Mediterranean (Flecha Max Q) and one Continental (Kentucky 31) tall fescue genotypes were used as control checks.

## Results

### Chloroplast sequence comparison, polymorphism and similarity index

Among 48 chloroplast primers, clear amplifications were found in 38 primers. The amplifications of the remaining 10 primers (NFTCHL 8, 10, 11, 12, 15, 16, 18, 21, 26 and 39) were not distinct, thus the PCR products of those primers were not sequenced. PCR products of five primers yielded poor quality sequences. Comparing 20 kb sequence reads among the 12 varieties/accessions, 167 polymorphic sites were identified, of which 129 were found to be SNPs, 25 were single base pair deletion, and 13 were multi-base indels which ranged from 3 to 47 bp (Table B in S1 File). Both morphotype and species-specific SNPs and indels have been identified in the 20 kb sequences. Among the pairwise comparisons of different groups presented in Table 2, *F*. *pratensis* versus Mediterranean displayed the highest number of polymorphic sites (117) and lowest similarity (0.30), followed by *F*. *pratensis* versus *F*. *mairei*. The lowest number of polymorphic sites (2) and highest similarity (0.99) was found between Rhizomatous versus *F*. *glaucescens*. The numbers of polymorphic sites for any of the Mediterranean and *F*. *mairei* versus any of the Continental, Rhizomatous, *F*. *pratensis* and *F*. *glaucescens* were found above 100, while polymorphic sites between Rhizomatous versus Continental, Rhizomatous versus *F*. *glaucescens* and Continental versus *F*. *glaucescens* were found to be five or less. Concomitantly, the number of polymorphic sites between Continental versus *F*. *pratensis*, Rhizomatous versus *F*. *pratensis*, *F*. *glaucescens* versus *F*. *pratensis* and Mediterranean versus *F*. *mairei* were found within a range of 42 to 57 (Table 2).

**Table 2:** Polymorphism and similarity among tall fescue morphotypes and related species in 20 kb chloroplast sequences. Polymorphism and similarity index was calculated between the two plant groups in each row.

Plant group 1	Plant group 2	Polymorphic SNPs	Similarity index
*F*. *glaucescens*	Continental	5	0.97
*F*. *glaucescens*	Rhizomatous	2	0.99
*F*. *glaucescens*	Mediterranean	100	0.40
*F*. *pratensis*	Continental	42	0.75
*F*. *pratensis*	*F*. *glaucescens*	44	0.74
*F*. *pratensis*	Rhizomatous	45	0.73
*F*. *pratensis*	Mediterranean	117	0.30
*F*. *mairei*	Mediterranean	57	0.66
*F*. *mairei*	Rhizomatous	101	0.40
*F*. *mairei*	Continental	102	0.39
*F*. *mairei*	*F*. *glaucescens*	102	0.39
*F*. *mairei*	*F*. *pratensis*	106	0.37
Continental	Mediterranean	103	0.38
Rhizomatous	Continental	5	0.97
Rhizomatous	Mediterranean	105	0.37

### SSR marker polymorphism

Multiple fragments were yielded by each of 144 SSR primers. The number of fragments ranged from one to 24 per primer pair, which made the total marker number 1,212 (Table C in S1 File). KY-31 had the highest number of markers present (360), followed by Texoma MaxQII (354). The lowest number of markers present was found in Rita (140). As a group, the highest number of markers present was found in Continentals (“Texoma MaxQII” and “KY-31”) followed by Rhizomatous (“Torpedo” with 290 markers). The lowest number of markers present was found in diploid *F*. *pratensis* (“Ensign” and “Rita”), followed by tetraploid *F*. *mairei* (PI283312 and PI283313 with 166 and 182 markers). Mediterranean varieties (“Flecha MaxQ,” “Prosper” and “Resolute”) and tetraploid *F*. *glaucescens* (PI28965 and PI289654) had markers present within the range of 238–274 (Table C in S1 File).

### Morphotype-specific molecular marker identification

From the chloroplast sequence alignment information, eight primers (NFTCHL01, NFTCHL05, NFTCHL17, NFTCHL33, NFTCHL34, NFTCHL43, NFTCHL45 and NFTCHL48) were identified, which amplified 13 indels in different Fescue varieties/accessions. Among them, four primers (NFTCHL17, NFTCHL43, NFTCHL45 and NFTCHL48) showing indel variations between Mediterranean and Continental morphotypes were chosen for further validation. The biggest 47 bp indel was shown in the sequence produced by the NFTCHL45 primer (Table B in S1 File). Primers NFTCHL17, NFTCHL43, NFTCHL45 and NFTCHL48 produced band sizes of 670, 503, 750 and 643 bp in Mediterranean and 638, 506, 703 and 651 bp in Continental morphotypes, respectively.

The presence of SSR marker alleles between the two morphotypes was not as consistent as the chloroplast markers. Thus, potential marker identification was done considering the number of discriminating alleles between the Continental and Mediterranean morphotypes. A total of 26 primers were found to have at least two allelic bands discriminating the two morphotypes (Table C in S1 File). Three of them (nffa090, nffa204 and nffa338) were selected based on prominent bands.

### Phylogenetic analysis and age estimation

**Fig 1:**
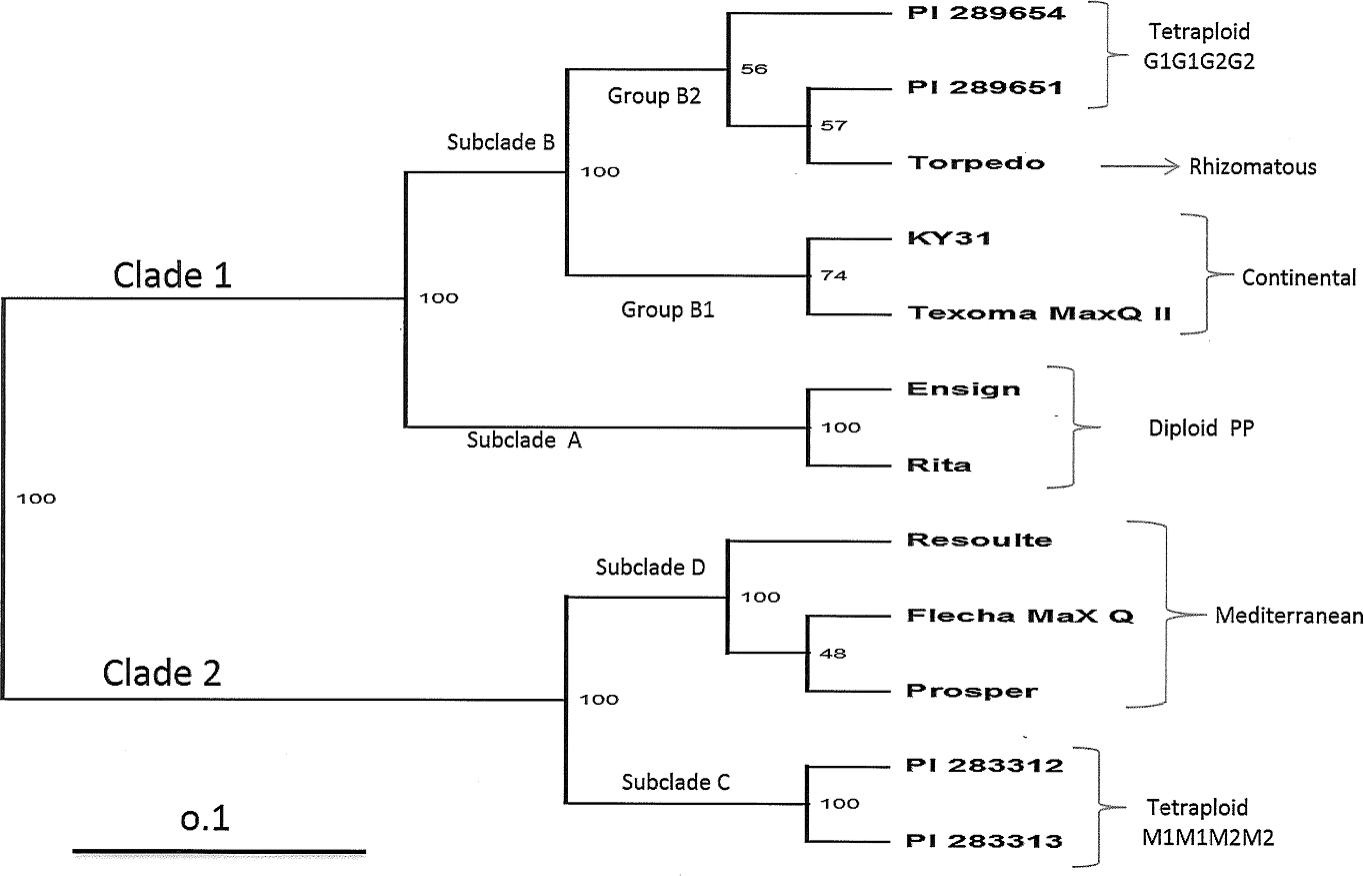
Phylogenetic relationship of 12 *Festuca* varieties/accessions using chloroplast sequences polymorphism obtained from 33 primer pairs designed from hypervariable regions of chloroplast genome. The values inside two horizontal bars depicts the per cent bootstrap values. The horizontal scale at bottom shows the Nei and Li/dice coefficients.

Geneious Pro 5.1 and FreeTree generated similar phylogenetic trees. The consensus tree was developed using polymorphic sequence information which resolved the *Festuca* species and morphotypes into two major lineages supported by a bootstrap value of 100 (Fig 1). Clade 1 consisted of Continental and Rhizomatous tall fescue along with diploid *F*. *pratensis* and tetraploid *F*. *glaucescens*. Clade 2 contained Mediterranean tall fescue along with tetraploid *F*. *mairei* (Fig 1). Clade 1 consisted of subclades A and B with 100% bootstrap value. Subclade A included both diploid *F*. *pratensis* cultivars (Ensign and Rita) with 100% bootstrap value. Subclade B was composed of two well supported groups (B1 and B2) with 100% bootstrap value. Group B1 resolved the Continental type (KY31 and Texoma MaxQ II, 74% bootstrapping), while group B2 resolved Rhizomatous and tetraploid *F*. *glaucescens* together with 56% bootstrapping value. Clade 2 was composed of two well supported subclades C and D with 100% bootstrapping value. Subclade C was resolved with two tetraploid *F*. *mairei* and subclade D was resolved with three Mediterranean morphotypes of tall fescue (Fig 1).

Like chloroplast sequence, both NTSYSpc and FreeTree produced similar phylogenetic trees using SSR polymorphisms. The phylogenetic map resolved the *Festuca* varieties/accessions into four major lineages comprising *F*. *pratensis* in clade 1, tetraploid *F*. *mairei* in clade 2, Mediterranean tall fescue in clade 3, and Continental, Rhizomatous and tetraploid *F*. *glaucescens* together in clade 4 (S1 Fig). Clade 1 was well supported with 100% bootstrapping value, but clade 2 was not (53% bootstrapping value). Clade 3 was also not well supported (75% bootstrapping value), but resolved the Mediterranean morphotype (Resolute, Flecha MaxQ and Prosper). Clade 4 was found to be comprised of two well supported (99% bootstrapping value) subclades (S1 Fig). Subclade A was resolved with Continental tall fescue (KY31 and Texoma MaxQ II), and subclade B was resolved with Rhizomatous (Torpedo) and tetraploid fescue (*F*. *Glaucescens*, PI 289651 and PI 289654).

**Fig 2:**
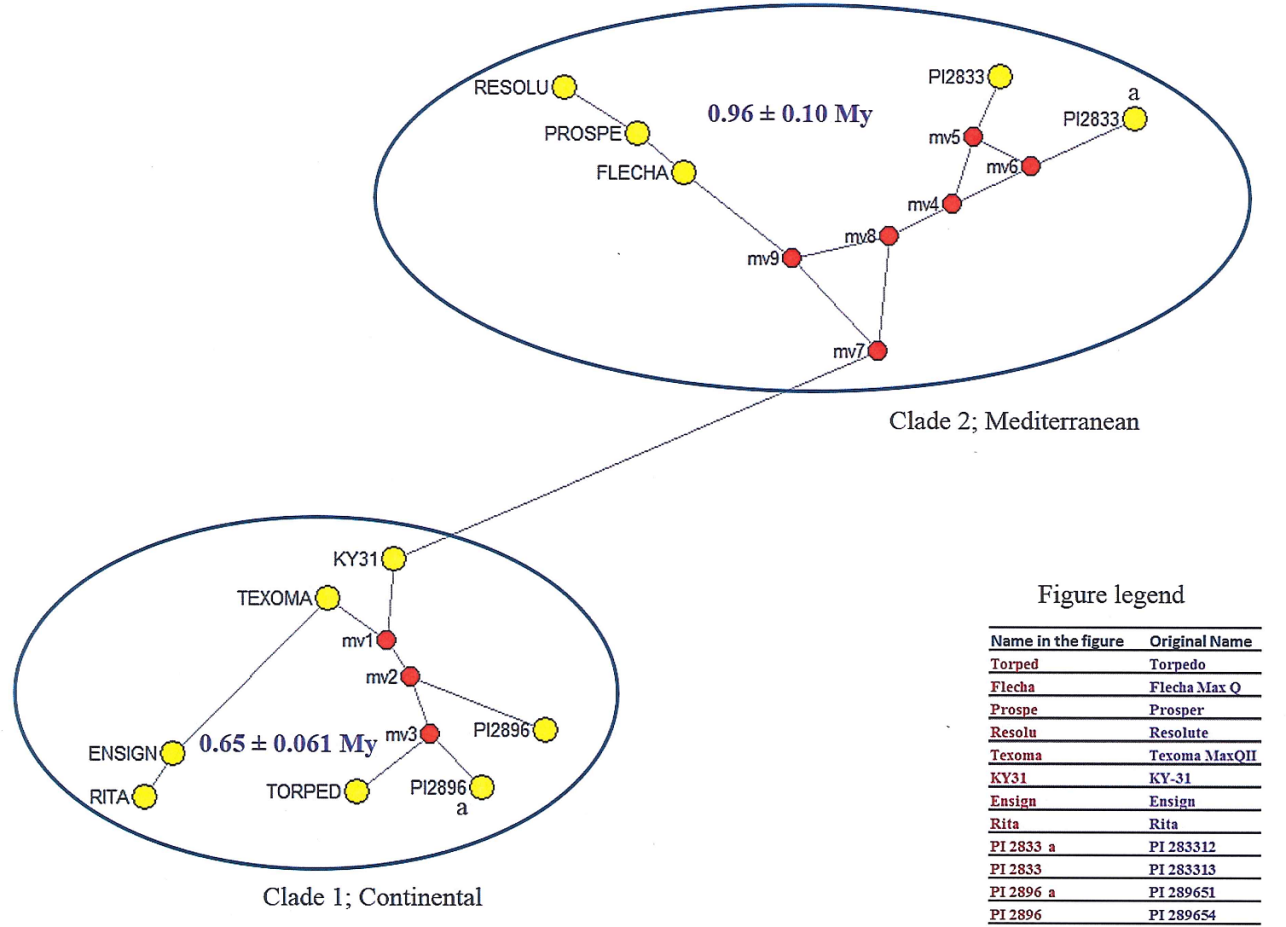
Median network formation for age estimation of diverged groups of 12 *Festuca* varieties/accessions. Red circles are the hypothesized median vectors to build the network with maximum parsimony.

Network analysis yielded two major clades which is exactly the same as the result of Free Tree analysis (Fig 2). Assuming mv1 as the ancestral node of clade 1, the mean genetic distances of the nodes’ 32.29 mutations with standard deviation (SD) = 3.04 provided the approximate age estimate of 0.65 ± 0.061 million years (My). Similarly, with mv7 as the ancestral node, clade 2 showed a mean genetic distance of 47.6 mutations with SD = 5 which provided the approximate age estimate of 0.96 ± 0.10 My. However, assuming a single origin for both clades and mv1 as a common ancestral node, clade 2 showed a mean genetic distance of 179.6 mutations with SD = 12.52 and an approximate estimated age of 3.62 ± 0.25 My. Considering mv7 as the common ancestral node, clade 1 showed a mean genetic distance of 163.71 mutations with SD = 11.87 and an estimated approximate age of 3.3 ± 0.24 My.

### Evaluation of morphotypes-specific markers

**Fig 3:**
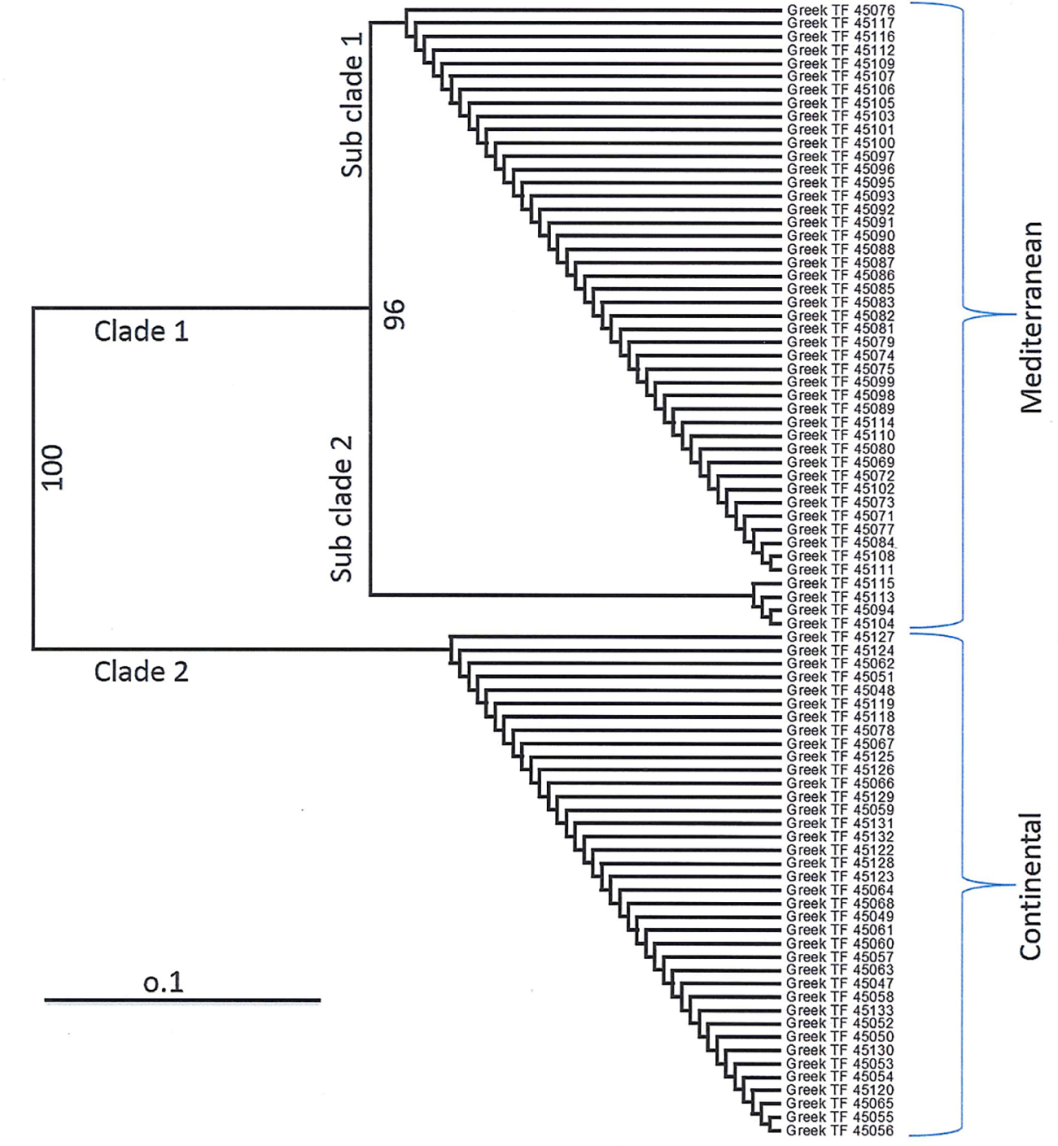
Classification of tall fescue germplasms collected from Greece based on four selected morphotype-specific chloroplast primers. The horizontal scale at bottom shows the Nei and Li/dice coefficients.

Chloroplast markers obtained from the selected four primers resolved the Greek collection of tall fescue into two major clades, recognizing clade 1 as Mediterranean morphotypes consisting of 47 accessions and clade 2 as Continental morphotypes consisting of 38 accessions. Clade 1 was subdivided into two conspicuous groups. One of the subgroups was found consisting of only four accessions and another group consisting of 43 accessions (Fig 3).

Three SSR primer pairs (nffa090, nffa204 and nffa338) were used for screening the Greek collection for validation. Two distinct groups were formed in the relationship analysis, based on polymorphism of the three primer pairs. Both groups were clearly separated from each other, comprising numerous sub- and sub-subgroups. Compared with the tree of chloroplast markers, accession 45104 was misplaced and joined the group of Continentals instead of Mediterraneans (S2 Fig).

**Fig 4:**
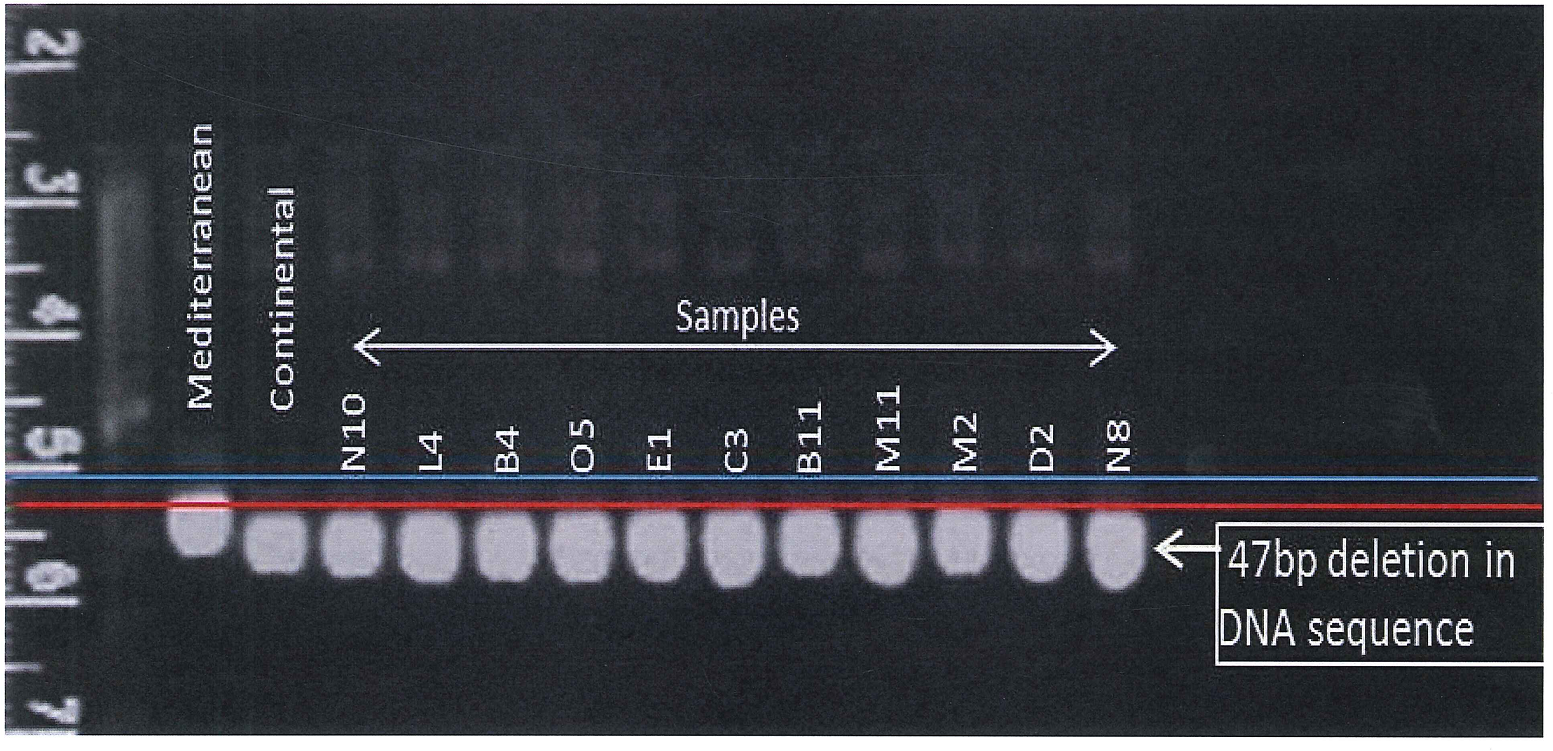
Morphotype determination of a tall fescue population grown in Oklahoma field with unknown origin using morphotype-specific NFTCHL45 chloroplast primer.

Screening ten interesting tall fescue plants from a population of unknown origin in an Oklahoma field using NFTCHL45 chloroplast primer resulted all to be Continental morphotype. All samples except the Mediterranean control produced a PCR band of 703 bp when amplified with NFTCHL45 primers, which is specific to the Continental morphotype (Fig 4).

## Discussion

Among the three morphotypes, Continental and Mediterranean tall fescue are mainly used as forage, and Rhizomatous as turf. Simultaneous improvement of both Continental and Mediterranean morphotypes is important as they have specific geographic niches and possess distinct phenotypes (summer active versus summer dormant). It is difficult to distinguish the morphotypes both visually and phenotypically, especially at the seedling stage, thus slowing the breeding process. Mediterranean tall fescue shows only partial summer dormancy. Large environmental influences have been recorded for the trait to be expressed [37]. Reliable phenotyping of Mediterranean tall fescue in the field is questionable. Development of morphotype-specific molecular markers and establishment of their relationships with other *Festuca* species can greatly help to establish an effective and reliable breeding program for the improvement of tall fescue.

### Chloroplast sequence comparison

Hypervariable regions of chloroplast DNA sequences were retrieved in this study by disregarding the conserved repetitive regions, because intergenic repetitive DNA with similar mutation rates was reported to have a lower contribution to evolutionary divergence than coding sequence [38–40]. Moreover, exon regions of coding genes were found exhibiting higher nucleotide diversity [41, 42]. Taking all this into account, 15 percent of the total chloroplast genome, mostly within the coding regions representing the most variable part of the genome, was used in this study. Eventually, we captured a significant amount of variability for morphogenetic relationship analysis and obtained reliable results which were then supported by SSR diversity.

### Morphotype-specific marker identification and evaluation

In comparing the chloroplast and SSR markers in this study, we found that a more accurate morphotypic group was yielded using chloroplast markers. It was evident from the banding pattern that there were unsynchronized allelic distributions of SSRs among the genotypes of Continental and Mediterranean accessions, while chloroplast primers produced allelic variation synchronized with the genotypes of all the varieties/accessions of each morphotype. A set of four chloroplast primers, NFTCHL17, NFTCHL43, NFTCHL45 and NFTCHL48 were found consistent in allelic variation between the two morphotypes. The validation of those primers using a tall fescue germplasm collection from Greece showed morphotypic classification completely in agreement with Takach, Mittal [24]. They classified the same collection using chloroplast matK sequence variability in the population. Thus, the use of these primers will be useful to distinguish between Continental and Mediterranean morphotypes of tall fescue. However, primer NFTCHL17 did not produce any allelic fragment in four of the Mediterranean accessions (Fig 3; Table D in S1 File). The same Greek collection was assayed using the selected SSR primers and similar result was obtained like chloroplast primer. One genotype was misplaced between the two groups, compared with the result of Takach, Mittal [24]. This result inferred the usefulness and accuracy of chloroplast markers for morphological classification, which completely agreed with the report of Cheng, De Vicente [43].

The chloroplast genome specific marker NFTCHL45 was used to identify tall fescue population with unknown origin, which have been established in Oklahoma for long time are expected to be Continental, because Mediterranean type is a recent introduction in the area. NFTCHL45 consistently amplified 750 and 703 bp products in Mediterranean and Continental genotypes, respectively. The PCR products can be easily resolved in agarose gel. Thus NFTCHL45 primers show high potential for using in marker assisted discrimination between Continental and Mediterranean morphotypes, which is very important for tall fescue breeding and improvement. Thus the marker has been provided to NRI Forage Analysis Core Service for the application of tall fescue morphotype discrimination. SSR markers were also very competent in distinguishing between the two morphotypes with very little error (<1.18%). Those markers will be extremely useful for screening early generation or advanced breeding lines.

### Morphotypic relationships using chloroplast DNA sequence and genomic SSR diversity

Various phylogenetic studies were conducted to explore the relationships among members of *Festuca* genus and its relatives using RFLP markers [44], EST-SSR markers [1], *matK* and *Acc1* gene sequence [5], and ITS-rDNA sequences [4, 5, 45, 46], mostly emphasizing Continental tall fescue. Several outlines of morphotypic and phylogenetic relationships of tall fescue was reported earlier [5, 45, 46]. Hand, Cogan [5] assessed the morphological relationship of hexaploid tall fescue using *matK*, *Acc1* and *CEN* individual gene sequences and reported a partial phylogenetic relationship among the morphotypes and progenitors. In the present study, by exploiting hypervariable regions of chloroplast genome sequence and SSR markers, we have captured a higher amount of variability in the chloroplast genome that has helped to identify molecular markers for marker assisted selection (MAS) in tall fescue breeding. The formation of two distinctly different groups in this study using chloroplast DNA sequences might be due to high genomic variability between Mediterranean and Continental tall fescue morphotypes [17, 47], which was in agreement with the result of Hand, Cogan [5], where they also reported a great genetic distance between the two morphotypes. Severe segregation distortion and imbalanced marker distribution was observed in a bi-parental mapping population developed using a Continental and a Mediterranean parent at NRI [48], which might be due to disrupted chromosomal pairing during population development. Chromosome pairing regulators must at least be disomic in doses in polyploid *F*. *arundinaceae* to confer a functional diploid like chromosome-pairing for stable disomic inheritance [49]. Based on *Festulolium* hybrid analysis, it was understandable that the chromosome pairing regulator gene resides on one of the sub genome of *F*. *glaucescens* in Continental tall fescue morphotype [50] It was reported that hybrid sterility between isolated tall fescue ecotypes resulted from irregular meiosis due to the breakdown of regulatory mechanism rather than chromosomal differentiation of the parental ecotypes [49] Thus, hybrid sterility between Continental and Mediterranean morphotypes might be the due to hemizygous ineffectiveness, meaning that at least one of the genome component of *F*. *glaucescens* should be different in Mediterranean morphotypes.

SSR markers demonstrated a very similar relationship among Mediterranean, Continental and Rhizomatous morphotypes like chloroplast sequence, with the exception of the formation of different and distant groups by *F*. *pratensis* and *F*. *mairei*. However, diploid *F*. *pratensis* was reported as a progenitor subgenome donor of the Continental morphotype and consistently formed groups/sister groups with Continentals [5, 14–16]. This variation in relationships shown by chloroplast and SSR marker systems suggests that SSR markers might be less reliable than chloroplast markers for a phylogenetic relationship study [43]. The underlying reason might be that, comparatively less variation is captured by the SSRs. Out of the 136 kb chloroplast genome, a comparison of 20 kb hypervariable regions must show a more reliable relationship than using only 144 markers in the 5–6 Gb haploid nuclear genome of tall fescue [51]. Moreover, the rate of developing genetic variation in the nuclear genome is much higher than the chloroplast genome because of the higher mutation rate [52]. Concomitantly, recombination is an additional source of genetic variation in the nuclear genome which makes the variation capture process harder than in the chloroplast genome.

The result of the morphological relationship using the chloroplast sequence demonstrated that Continental and tetraploid *F*. *glaucescens* (G1G1G2G2) along with Rhizomatous morphotypes formed sister groups to each other, whereas Rhizomatous and *F*. *glaucescens* were too close to be resolved. This indicated that, though Rhizomatous and Continentals are characterized as independent morphotypes [5, 12], both morphotypes and the tetraploid *F*. *glaucescens* might have shared genome in the evolutionary process. However, *F*. *glaucescens* was already reported as a genome donor of both Continental and Rhizomatous tall fescue [5, 13, 15]. Not only that, but a very low number of polymorphic sites in the chloroplast sequence (Table 2) among these three groups also suggested that Continental, Rhizomatous and *F*. *glaucescens* might have been evolved through the same maternal inheritance.

Similarly, subclade B consisting of Continental, *F*. *glaucescens* and Rhizomatous tall fescue formed a sister group with subclade A consisting of *F*. *pratensis*, suggesting that Continental, Rhizomatous and *F*. *glaucescens* have an equally close relationship with *F*. *pratensis*. This observation was supported by previous reports, where it was proposed that *F*. *pratensis* might be closely associated with hexaploid tall fescue evolution [5, 15, 53]. The sister group formation of Mediterranean genotypes with the tetraploids of *F*. *mairei* under a different clade suggests that *F*. *mairei* is closer to Mediterranean morphotypes than *F*. *pratensis* and *F*. *glaucescens*. This part of our result is not fully in agreement with the statement that *F*. *pratensis* and *F*. *glaucescens* are the genome donor of all hexaploid tall fescue [5, 44, 54]. The chloroplast sequence comparison between Mediterranean and *F*. *mairei* displayed almost half the polymorphism and double the similarity of Mediterranean versus *F*. *pratensis* and Mediterranean versus *F*. *glaucescens* (Table 2). At the same time, the polymorphism and similarity index between Mediterranean and *F*. *mairei* was very similar to the polymorphism and similarity index between *F*. *pratensis* versus Continental, *F*. *pratensis* versus Rhizomatous and *F*. *pratensis* versus *F*. *glaucescens* (Table 2). Both phylogenetic grouping and chloroplast sequence polymorphism, and similarity index results inferred the idea that *F*. *mairei* might have an evolutionary relationship with Mediterranean tall fescue comparable with the relationship which has been found between *F*. *pratensis* versus either one of the Continentals, Rhizomatous and *F*. *glaucescens*. Earlier reports suggested *F*. *pratensis* as an evolutionary member of *F*. *glaucescens* [13, 14], Continentals and Rhizomatous tall fescue [5, 13, 15]. Based on this result, it is also possible that *F*. *mairei* might be a progenitor of the Mediterranean morphotype. Various studies suggested Mediterranean and Continental morphotypes might be genetically very distant and evolved through independent evolutionary events [5, 17]. Simultaneously, *F*. *mairei* was also previously reported genetically very close to hexaploid tall fescue [55]. Based on hybridity study, Borrill [26] also suggested *F*. *mairei* might be a probable progenitor of a polyploid race of *Festuca sp* predominant in Morocco. He discussed various options of polyploid formation and proposed that the polyploids consisting of xeromorphic characters of *F*. *mairei* might have survived and persisted under arid climatic conditions in northwest Africa. Not only the variation of polymorphic SNPs and similarity index, but six insertion/deletion events out of a total of 13 were found in both the Mediterranean morphotype and *F*. *mairei*, which were absent in other groups. This also signifies the idea of *F*. *mairei* being a progenitor of Mediterranean tall fescue because, insertion, deletion and inversion events in the chloroplast genome were found extremely useful to resolve the phylogenetic relationship of higher plants [56, 57].

In the network analysis, no median vector in Clade 1 for central node formation to connect clade 2, due to the existence of high sequence dissimilarities, indicates independent origins of the two clades which was proposed earlier [5, 58]. The divergence estimates also implied independent colonization of the two clades at two different times. The dating of the Mediterranean clades (0.96 My) is in agreement with the dating of the most recently evolved Mediterranean ryegrasses (*Lolium perenne*/*L*. *rigidum*; 1 My), Mediterranean red fescue (1.6 My) and Neozeylandic red fescue (1.0 My) [3]. Considering the single ancestral node (either of mv1 and mv7), both clades 1 and 2 could be dated back to 3.3 My and 3.6 My, respectively, meaning that the clades’ members were equally important for colonization of *F*. *arundinacea*. Moreover, the dating estimates in this study are in agreement with previous dating estimates (i.e., *F*. *arundinacea*, c. 3.5 My) of Inda, Segarra-Moragues [3]. They also showed that all the broad leaf *Schedonorus* lineage which includes *L*. *perenne*, *L*. *rigidum*, *L*. *canariense*, *F*. *pratensis*, *F*. *arundinacea*, *F*. *letourneuxiana and F*. *mairei were* colonized in the Mediterranean region. The dispersal of Continentals (*F*. *pratensis* and *F*. *arundinacea*) occurred from circum-Mediterranean refugia towards northern latitudes [3], thus the early evolvement of Mediterranean morphotypes is likely.

It was clear from the discussion that *F*. *mairei* is a probable progenitor of the Mediterranean morphotype. Alongside, Mediterranean group has evolved earlier than Continental; thus the group itself might be denoted as a different species of hexaploid tall fescue under *Festuca* genus rather than a morphotype.

## Conclusion

Four chloroplast and three SSR primers were identified in this study which can clearly discriminate Continental morphotypes from Mediterranean. These markers will be highly useful in the breeding program as a tool to improve tall fescue for increasing persistence, summer stress tolerance and productivity. Based on the age estimation and phylogenetic grouping, there is an indication that Mediterranean tall fescue might have evolved earlier than Continentals and *F*. *mairei* could be a probable progenitor of Mediterranean-type tall fescue. However, this information warrants further exploration to confirm the genome structure of Mediterranean tall fescue using various cytological and phylogenetic analyses of all the potential progenitors

## Supporting information

S1 Fig Phylogenetic relationship of 12 *Festuca* varieties/accessions using polymorphic SSR markers. (TIF)

S2 Fig Phylogenetic classification of a tall fescue germplasm collection from Greece based on polymorphism of three selected SSR primers. (TIF)

S1 File Accommodation of Table A-D. Table A; Primers developed from the hypervariable regions of chloroplast genomes and amplified in 12 *Festuca* varieties/accessions. Table B; SNPs, insertion and deletion polymorphisms among the retrieved sequences of 12 *Festuca* varieties/accessions in compare to the reference chloroplast genome of tall fescue. Table C; SSR genotyping results across 12 *Festuca* varieties/accessions with all fragment information. Table D; Genotyping results of the selected chloroplast and SSR markers in the collected population (Greek collection) used for validation.
(XLSX)
