# Supplementary figures and images for: Molecular discrimination of tall fescue morphotypes in association with Festuca relatives
Source: PLoS One. 2018 Jan 17;13(1):e0191343. doi: 10.1371/journal.pone.0191343 (PMC5771633; doi:10.1371/journal.pone.0191343)

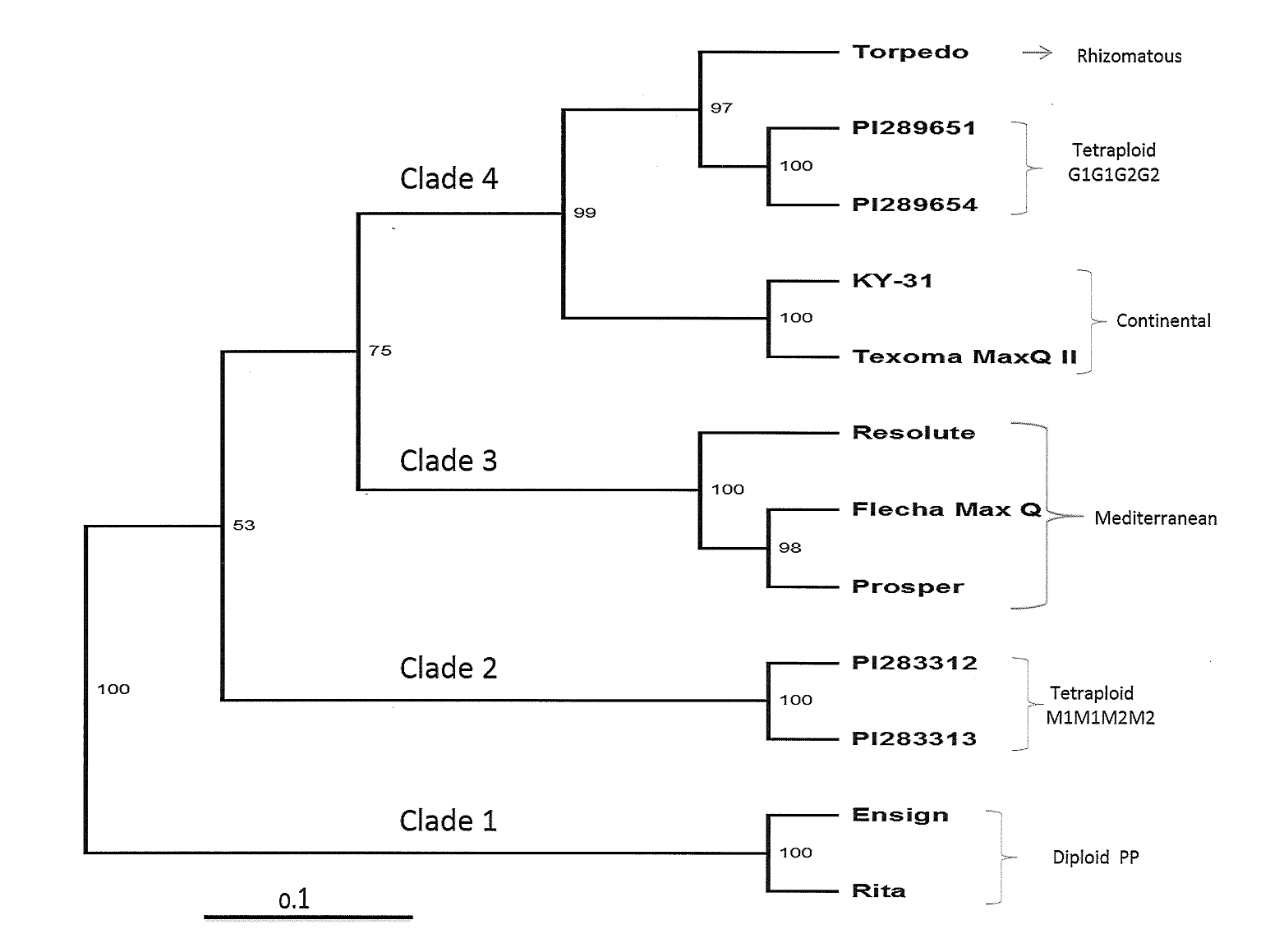

Supplement: S1 Fig — (TIF) [file pone.0191343.s001.tif]

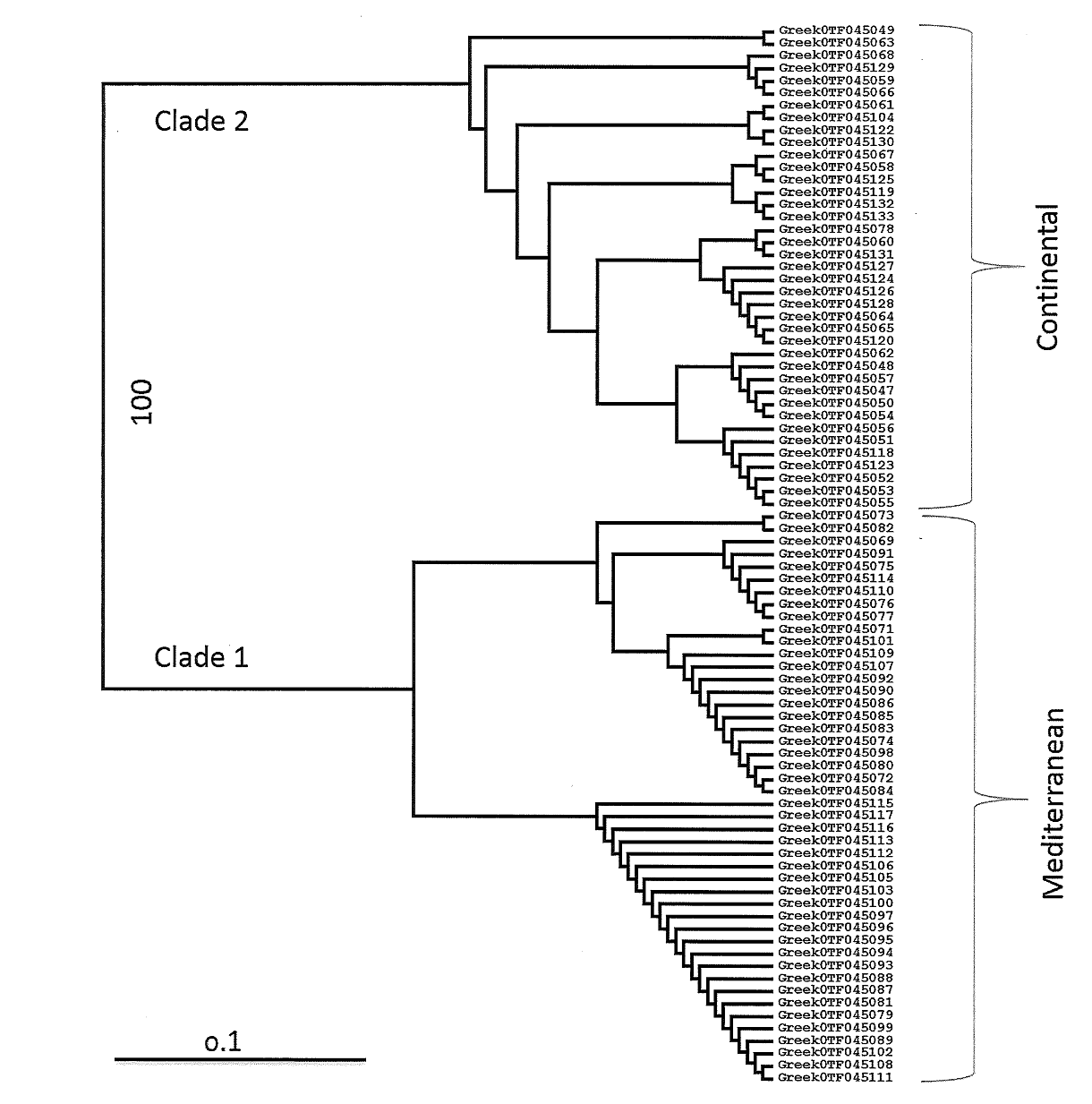

Supplement: S2 Fig — (TIF) [file pone.0191343.s002.tif]
